# Supplementary material for: Identification of pattern mining algorithm for rugby league players positional groups separation based on movement patterns
Source: PLoS One. 2024 May 1;19(5):e0301608. doi: 10.1371/journal.pone.0301608 (PMC11062535; doi:10.1371/journal.pone.0301608)
Supplement: Attachment — Submitted filename: Response to Reviewers.pdf [file pone.0301608.s001.pdf]

Emily Chenette
Editor-in-Chief
PLoS ONE

12 February 2024

Dear Emily Chenette,

We would like to express our appreciation for the thorough review of our manuscript titled "Identification of pattern mining algorithm for rugby league players positional groups separation based on movement patterns." We are grateful for the time and effort invested by the reviewers in evaluating our work and providing constructive feedback.

We have carefully considered the comments and suggestions provided by the reviewers and have made several revisions to address their concerns. Below, we provide detailed responses to each of the reviewers' comments:

Reviewers' Comments	Authors' Response
Reviewer #1:<br>This article applies three different pattern mining algorithms to private rugby league data, using the output of these algorithms with five different machine learning classification algorithms to determine whether the different movement patterns could be used to distinguish between hookers and wingers. A more complete description of the data used would be welcome (e.g., how many teams were represented? How much playing time did the players have? How was data cleaned and were there any exclusion criteria?), but the described experiments seem sound and the conclusions could be beneficial in other related areas.<br><br>Some more minor recommendations include:<br>1) In the "Data and Processing" subsection of the Method, Figure 1 is referenced, when it should be Table 1.<br><br>2) In "Separation of Players into Playing Position", "set unique of movement patterns" should be "set of unique movement patterns".<br><br>3) Table 3 mentions that "default" parameter values were used for some of the tested algorithms. Please move the detail that scikit-learn was used to the sentence that references this table so it is understood that it is the defaults from this library that are used.<br><br>4) In "Overlapped movement patterns between positions per algorithm", "based on often" should be "based on how often" (a few instances of this)<br><br>5)Table 1 describes 10 unit movement characters, but several more appear to be used later in the paper. A description of all unit movement characters should be included.<br><br>6) A figure is referenced as Fig ??<br><br>7) in the "Discussion"* "noteworthy to point out" seems redundant - replace with "noteworthy"	A complete description of the data used is presented in the Method section under the "Overview" and "Data and Processing" subsections.<br><br>Some more minor recommendations include:<br>1) Thank you for spotting this error, it is now fixed. The error was due to referencing while formatting the manuscript using OverLeaf.<br><br>2) Thank you for spotting this error, it is now fixed.<br><br>3) Thank you for this suggestion. The sentence that included the details of scikit-learn was used is moved and now precede the sentence with details of parameter values.<br><br>4) Thank you for spotting this error, it is now being rectified.<br><br>5) Thank you for this suggestion. Table 1 is an example of processing GPS data into movement sequence following existing framework published by [15] and do not contain all movement unit character as it is merely for illustrative purpose. All movement unit characters and methods to process GPS data into movement sequences are published in [15].<br><br>6) The figure referenced as Fig ?? is present in the discussion section and was due to transferring the manuscript from OverLeaf latex to Microsoft Word document. Thank you for spotting it and it is now fixed.<br><br>7) "Noteworthy to point out" is now replaced with "noteworthy".
Reviewer #2:<br><br>GENERAL COMMENTS<br><br>The authors have presented an analytics-focused study examining the performance and similarity of identifying movement patterns in rugby. The methods are well justified based on previous pattern classification works, and the idea of comparing algorithms is sound. Where I see the major weaknesses of the paper in present form is in the results and discussion, namely: (1) the readability	We thank this reviewer for his detailed (i.e., general and specific) comments. As suggested in the reveiwer's general comment, this study is both data and analytics focused study. However, the study was motivated from sport practitioners' perspective with questions regarding the best type of movement patterns to be use for sport performance analytics, given the different obtainable types of patterns.<br>As such, we strived to provide a robust analysis that answered different practical viewpoints (e.g., Is there similarity among distinct obtainable patterns? Is the movement similarity between tactical positions known to be

and interpretability of many of the results presented is not easy or intuitive (see specific comments for this section below); and (2) the practical implications of what was found (e.g. lack of similarity in algorithms) is potentially not discussed in enough detail. I think once addressed this paper can progress understanding in this area and provide some useful recommendations for future pattern mining and classification work in sport.<br><br>I recently received a review on one of my own papers where the reviewer provided an 'about me' section, which I found quite valuable in placing additional context around their comments, so it is something that I am trying to add to my own reviews:<br>I have a research background primarily in sports injury and performance biomechanics, but have a secondary research focus on using data and analytics approaches to characterise and improve sports performance. My work in this area has predominantly focused on netball, with a little in Australian football. I believe I have a basic understanding of the algorithms and approaches used in this paper – but wouldn't call myself an expert in all of these.I am also open to being identified on papers I reviewed – so am signing off my review here. Aaron FoxCentre for Sport Research, Deakin University Please see below for specific comments. As no line numbers are present on the submission I've tried to use the page numbers and rough section on the page, or used a quote to indicate where the comment relates to.	different? Which movement patterns best separate players into playing positions? among others) and discussed the results (in sections and subsections) considering all viewpoints.<br>We understand analytics-focused study can be quite difficult to follow for sports audience and that was why the study was simplified into three vis-à-vis (1) quantification of sets of distinct movement pattern similarity, (2) investigation into the overlap of movement patterns across obtainable types, and (3) use of machine learning to quantify how accurate movement patterns of each type can separate players into two tactical positions, known by practitioners to be different. This was mentioned in the last paragraph of the introduction.<br><br>We are grateful to receive such elaborate review of this work. The feedback provided were great and help shaped the presentation of this study.
SPECIFIC COMMENTS<br>Abstract<br>In the abstract it is a little unclear how the "best set of movement patterns" is identified based on the three algorithms. I'm expecting more in the main methods, but some indication in the abstract on how this is evaluated would be useful.<br><br>Similarly, it is stated that "AprioriClose…shared no significant similarity" with the other algorithms – but it's unclear how this was determined. Including the Jaccard score here between these algorithms would provide a contrast to the other value presented in the prior sentence. I think this is particularly relevant to establish, as the Jaccard score for the previous comparison (i.e. 0.19) isn't exactly high for this metric.<br><br>Prior to mentioning the results from the Multi-layered perceptron algorithm, this and it's associated metrics (i.e. precision, recall, F1 score) are not mentioned in the abstract – so it sort of comes out of the blue. Presumably these relate to the classification algorithms mentioned earlier in the abstract – but some additional clarity around these methods, so that the subsequent results make sense in the abstract would be useful.	**<u>Specific Comments</u>**<br>Abstract<br>The "best set of movement patterns" is identified based on the three algorithms because each represent one of the three obtainable types (i.e., consecutive, non-consecutive, and non-sequential) of movement patterns. The abstract is now revised to highlight the three types of obtainable movement patterns.<br><br>The Jaccard similarity scores are now added to provide contrast to the other values.<br><br>The sentence "… and machine learning classification algorithms were used to identify which algorithm's movement patterns is the best to separate playing positions" imply the use of some classification algorithms for modelling and then its evaluation.<br>Due to the number of words constraints, we can not list all five algorithms as well as mentioned the performance evaluation metrics for classification modelling utilised in the study beforehand.
Introduction<br>The introduction provides a good summary of the area and justification for the study. I thought that there could be more of an emphasis on the practical justification of the study (i.e. why movement patterns are important) over the detailed analysis of the pattern mining algorithms – but can potentially see the need for this given the study is more so focused on algorithms over practical implications in rugby at this point. I would still consider shifting the emphasis slightly – but would leave this up to the authors.<br><br>Page 2/16: "However, the insights are currently based on aggregated physical, technical and tactical demands either across a whole match or for specific periods within the game." A follow-up sentence to this explaining why this is an issue would help set-up the subsequent notion of investigating pattern mining algorithms. This is somewhat touched on later (on page 3/16), but I feel that this statement would work better up here.	Introduction<br>The practical justification of the study (i.e. why movement patterns are important) was provided (i.e., third sentence of the second paragraph) before the analysis of pattern mining algorithm.<br><br>The explanation of why this is an issue was presented, in the same paragraph but before this highlight sentence, in the context of performance indicator limitation.<br>We have now revised and moved the sentence, "Each performance indicator typically accounts for a single activity or event (e.g., pass, shots, total distance covered, average match speed) and has been widely used to derive actionable insights for making data-driven decisions", after the "However, the insights…." sentence.<br>Also, we continued to touch on it later (on page 3/16) by discussing the strength of movement patterns in comparison to traditional performance indicators.<br><br>Relevant literature are now cited to support this statement.

Page 2/16: "Nowadays, sequential pattern mining algorithms are applied to sports data to profile players' movements." – some citation to relevant literature to support this would be nice.	
Page 2-3/16: "Also, the algorithm was reportedly developed because the existing Closed Contiguous Sequential Pattern mining (CCSpan) algorithm may not produce usable movement patterns for sporting contexts and could not scale well on large sets of (lengthy) players' movement sequences." – I'm a big fan of active writing and taking ownership over the previous work a group of authors has conducted. Given this is past paper you're referring to is your work, I'd recommend referring to it in this way (i.e. rather than "was reportedly developed…" – consider "we developed…").	We have now taken ownership of our work and rewrite the sentence based on the recommendation. Thank you for suggesting active writing.
Page 3/16: "However, the investigation of which type of movement pattern is best for rugby league players' movement profiling is yet to be explored" – should this read "which type of movement pattern mining algorithm is best…"?	From a practical viewpoint, "However, the investigation of which type of movement pattern is best for rugby league players' movement profiling is yet to be explored", is best as practitioners are interested in the movement patterns and not necessarily the algorithm.<br>On the other hand, "… which type of movement pattern mining algorithm is best…", suits the algorithm aspects of this study. Such is this study – the strive to balance practical and algorithmic aspects!<br>We have revised this sentence as "However, the investigation of which type of movement pattern (mining algorithm) is best for rugby league players' movement profiling is yet to be explored.
Methods<br>Given there are numerous aims and methods associated with these, I think the back-end of the introduction could use some more solid statements around what these specific aims are (e.g. "The specific aims of this study are (1) …; (2) …"). This could then link into the 'overview' section of the methods by linking up these aims to the various approaches described (i.e. "For aim 1…we did this"; for aim 2…" etc). I feel this would help with clarity around what the various algorithms and metrics are being used for.	Methods<br>There is only one overarching aim of this study – identify which type of movement pattern (mining algorithm) is best to profile players into two distinct rugby league playing positions (i.e., hookers and wingers). The achievement of this aim is based on three steps. This is now included in the last paragraph of the introduction and iterated in the "Methods" section.
Page 4/16: "A total of 1,036 total observations were included which represent players' movement sequence per fixture." Some details (perhaps as supplementary info) around how the players data is spread across these observations could provide additional context on the dataset (e.g. presumably the 22 hookers didn't play every game? Or maybe they did?).	The sentence is now revised, "A total of 1,036 total observations (hookers = 500 and wingers = 536) were included which represent players' movement sequence per fixture.", and included the spread of the players.<br>Also, the classification data which include the players' spread is now added as a supplementary info.
Page 5/16: "The studies [12, 17] used similar parameter values to enable the extraction of large and longer-length frequent patterns." It would help to clarify the sports these are in – it looks like they are both rugby? Which would provide further support for replicating these methods.	The sports are now included. Study [12] is a based on rugby union data and study [17] is based on rugby league data.
Page 5/16: "a set of unique movement patterns was derived by computing the union of all sets of extracted movement patterns." I'm a little unclear of what this refers to/how it was done. Some extra detail on these methods would help others replicate these analyses.	Thank you for this comment. Union is a basic set operation in mathematics/computing, which we assumed readers would understand. It is simply concatenating sets of items into a single set that retains only unique items. We have now introduced the word "mathematical" as an adjective to the word union in the same sentence.
Page 6/16: The section label (and subsequent use of this terminology) 'Pattern Mining Algorithms (Movement Patterns) Validation' – with particular reference to the use of 'validation' – seems slightly incorrect to me. To validate these pattern mining algorithms, you would need to know what the 'real' patterns are, whereas it seems the paper assumes that the algorithms are simply extracting the true or real patterns. 'Agreement' seems like a better term over 'validation.'	We agree with the inaccurate use of the word "Validation" and "validate" in this section and through out the article. Hence, the whole text is revised and does not contain those word.<br>This section is now labelled as "Selection Analysis of (Movement) Pattern Mining Algorithms", which is a more precise and suitable title.
Page 6/16: "For each pair, the most frequent-50 and least frequent-50 extracted movement patterns of each pattern mining algorithm were checked for overlapping and visualised." Why the most and least frequent 50? Why not more or less? This seems a little subjective and could use some further justification.	Practitioners are most interested in the top or bottom 10 items in any given list. And given the lowest number of patterns extracted by one of the algorithms (i.e., AprioriClose – 155), we investigated overlap based on 1/3 patterns of the lowest set of extracted patterns which is 51.7 (and 50 seems more subjectively appropriate).

	The statement, "For each pair, the most frequent-50 and least frequent-50 extracted movement patterns of each pattern mining algorithm were checked for overlapping and visualised.", is now revised.
Page 7/16: "The cross-validated models' performances were evaluated by aggregated accuracy, precision, recall and f1-score metrics" – it's unclear if this was done on a separate 'test' dataset outside of the training set. If it wasn't, then this needs to be clear – and perhaps it's worth considering doing so to truly validate these models on 'unseen' data.	In each fold of a cross-validation process, there is a separate 'test' dataset outside of the training set. Since, we use 10-fold cross validation, the performance results are based on aggregated measures which were obtained from separate test set within each fold.<br>This is now clear stated out in the manuscript.
Results<br>Currently the main issue I see with the results is the difficulty in following some ideas due to the lack of intuitiveness in interpreting the results. When I saw the word-clouds as figures in the PDF document, I initially thought they were a watermark-like placeholder and I needed to download the figure – but once I had downloaded the figure I realised what they were. These word-cloud figures aren't easily interpretable (particularly given that out of context, the text strings don't really mean anything) – so I think a different approach to presenting these would be appropriate. If keeping some sort of visual representation of movement patterns is desired (see my next comment on this though), it would be nice if you could make these visuals more intuitive and combine a lot of this information into one figure/table (i.e. combining Fig. 2-6 to show overlap across the three algorithms and when split by position). I don't think this is an easy task, and may even be unachievable (but see my next comment for why I don't think this matters).	Results<br>The word-clouds are summative visual representation of movement patterns and its frequency.<br>Fig. 2 -6 cannot be combined as they illustrate and communicate different results. Even if presented in a table, there would separate table for each result.
Similar to above, I don't necessarily think the entire sections on comparing more individualised movement patterns between algorithms adds that much. The idea behind metrics like the Jaccard index is that it provides this summary-style quantitative metric to demonstrate similarity – which is easy to understand. Probing through individual pattern similarities like is done across most of the results section is quite difficult to follow and interpret meaning from. If you wish to probe these more individualised comparisons, I strongly suggest finding a more summary-like way of doing this to make these comparisons more digestible for readers. I think this idea is demonstrated in your summary paragraph at the beginning of the discussion – where you state "Overall, the findings suggest that the LCCspm pattern mining algorithm provided the best set of movement patterns for separating hookers and wingers and that there is a lack of similarity in the extracted movement patterns between algorithms" – which I'd argue has been predominantly determined based on the Jaccard score and classification models (i.e. these main findings haven't really been determined based on individual movement pattern comparisons).	As useful and summary-style the Jaccard similarity score metric is, it does not show readers and practitioners the movement patterns that is being discussed. Therefore, the illustration of movement patterns became paramount.<br><br>The comparison of movement patterns between algorithm and their visualisation is necessary as it communicate the actual patterns. Additionally, some of movement patterns were decoded in the text.<br><br>As noted, the summary paragraph at the beginning of the discussion served it purpose of summarising the results presented in the study.<br>However, the visuals and results reported in the result section were made comprehensive and robust enough to benefit readers who are interested in text, tables and or charts.
Similar to the figures, I'm not sure how much added context Table 6 adds given that the pattern indicators aren't intuitive to readers – i.e. "eeeeefeeeeeeeee" could be the noise someone makes when dropping something heavy on their toe. Based on this I think Table 6 could be removed.	Table 6 added context to the classification modelling results. It revealed the top contributing movement patterns used as variables by the classification model. As you noticed, the movement pattern "eeeeefeeeeeeeee" extracted by the SMP framework is a long sequence of activities and not part of the overlapped movement patterns presented in the other subsections of the result section. The table cannot be removed.
Discussion<br><br>Page 12/16: "More so, Jaccard similarity scores (1 being full similarity) ranged from 0.008 to 0.19 among movement patterns algorithms (Table 4), suggesting a lack of similarity in the extracted patterns overall. LCCspm and LCS sets of movement patterns shared higher similarity because both algorithms extract some form of sequential movement patterns as opposed to AprioriClose non-sequential patterns. Based on these results, the LCCspm algorithm is justified and validated as the best for profiling movement patterns of rugby league players into hookers and wingers playing positions."	Discussion<br><br>The implication of the difference of applying different pattern mining algorithm to obtain different type of movement patterns is already discussed in the introduction section.<br>Nonetheless, the discussion about the lack of similarity between algorithms and the explanation of why such difference happened is now included as it elaborates on the results.

I think further discussion on the lack of similarity between algorithms is worthwhile. Even the Jaccard index of 0.19 is relatively low, and this probably has implications in research and practical applications where different algorithms are used. For example, two studies that use different algorithms could come to completely different conclusions on what the movement patterns in players of a given sport are. Some further explanation of why these differences happen, and discussion of these implications would be relevant. The importance of this links into a later statement on page 13/16 – "Applying pattern mining algorithms to uncover the sequential nature of the occurrences of activity enables the better capability to classify positional groups and aid in enhanced training specificity." With different algorithm use, these identified patterns will likely differ, which might impact on this training specificity you refer to.<br><br>Page 13/16: "This further validates LCCspm algorithm and closed contiguous movement patterns as the best pattern to profile rugby league players into playing positions." I'm not sure the preceding information here links to this statement – i.e. I don't think the features included as more important in these models supports one as being better over the other, only the classification accuracy can really be used to support this.	The classification accuracy is used to support LCCspm as being best over others. Fundamentally, the features extracted by LCCspm and used for such classification determined the accuracy of the model. Hence, the ability of LCCspm to extract closed contiguous movement patterns ensures it being the best movement pattern mining algorithm to profile players into positional group.<br>The sentence is now revised.
Reviewer #3:<br><br>Thank you for the opportunity to review this well written manuscript.<br>I have only minor revisions/questions to be answered before I believe this manuscript is acceptable for publication.<br><br>1. Could different cohorts e.g., southern Hempishere or women's players be incorporated?<br>2. How can the results of your study be used practically? e.g. can you add more detail into the discussion regarding how practitioners can apply your findings?	Thank you for your overall comments.<br><br>1) Since rugby league is a physically intensive sport and the demand of match game on players across aspects (i.e., different pathways, competitions, and categories) is already established, it would be better not to incorporate players of known different match demands into the same investigation. Rather, investigation into the best movement patterns to profile players that participated in the same competition or pathway or category should be conducted – this is what was implemented in this study.<br><br>2) One of the overall findings of the study revealed LCCspm algorithm provided the best movement patterns to profile players into distinct playing positions. This practically means that sports performance analyst or data scientist should extract closed contiguous movement patterns for player profiling when conducting various types of analysis.<br>This is now appended to the discussion.
